# Progression of Myopia in School-Aged Children After COVID-19 Home Confinement

**DOI:** 10.1001/jamaophthalmol.2020.6239

**Published:** 2021-01-14

**Authors:** Jiaxing Wang, Ying Li, David C. Musch, Nan Wei, Xiaoli Qi, Gang Ding, Xue Li, Jing Li, Linlin Song, Ying Zhang, Yuxian Ning, Xiaoyu Zeng, Ning Hua, Shuo Li, Xuehan Qian

**Affiliations:** 1 Department of Ophthalmology, Emory University, Atlanta, Georgia; 2 Department of Ophthalmology and Visual Sciences, Department of Epidemiology, University of Michigan, Ann Arbor; 3 Department of Strabismus and Pediatric Ophthalmology, Tianjin Medical University Eye Hospital, Tianjin, China; 4 Department of Respiratory and Critical Medicine, Tianjin Medical University General Hospital, Tianjin, China

## Abstract

**Importance:**

Time spent in outdoor activities has decreased owing to home confinement for the coronavirus disease 2019 (COVID-19) pandemic. Concerns have been raised about whether home confinement may have worsened the burden of myopia owing to substantially decreased time spent outdoors and increased screen time at home.

**Objective:**

To investigate the refractive changes and prevalence of myopia in school-aged children during the COVID-19 home confinement.

**Design, Setting, and Participants:**

A prospective cross-sectional study using school-based photoscreenings in 123 535 children aged 6 to 13 years from 10 elementary schools in Feicheng, China, was conducted. The study was performed during 6 consecutive years (2015-2020). Data were analyzed in July 2020.

**Exposures:**

Noncycloplegic photorefraction was examined using a photoscreener device.

**Main Outcomes and Measures:**

The spherical equivalent refraction was recorded for each child and the prevalence of myopia for each age group during each year was calculated. The mean spherical equivalent refraction and prevalence of myopia were compared between 2020 (after home confinement) and the previous 5 years for each age group.

**Results:**

Of the 123 535 children included in the study, 64 335 (52.1%) were boys. A total of 194 904 test results (389 808 eyes) were included in the analysis. A substantial myopic shift (approximately −0.3 diopters [D]) was found in the 2020 school-based photoscreenings compared with previous years (2015-2019) for younger children aged 6 (−0.32 D), 7 (−0.28 D), and 8 (−0.29 D) years. The prevalence of myopia in the 2020 photoscreenings was higher than the highest prevalence of myopia within 2015-2019 for children aged 6 (21.5% vs 5.7%), 7 (26.2% vs 16.2%), and 8 (37.2% vs 27.7%) years. The differences in spherical equivalent refraction and the prevalence of myopia between 2020 and previous years were minimal in children aged 9 to 13 years.

**Conclusions and Relevance:**

Home confinement during the COVID-19 pandemic appeared to be associated with a significant myopic shift for children aged 6 to 8 years according to 2020 school-based photoscreenings. However, numerous limitations warrant caution in the interpretation of these associations, including use of noncycloplegic refractions and lack of orthokeratology history or ocular biometry data. Younger children’s refractive status may be more sensitive to environmental changes than older ages, given the younger children are in a critical period for the development of myopia.

## Key Points

**Question:**

Is home confinement due to coronavirus disease 2019 associated with the burden of myopia?

**Findings:**

In this cross-sectional study that included 194 904 photoscreening tests conducted in 123 535 children, a substantial myopic shift (−0.3 diopters) was noted after home confinement due to coronavirus disease 2019 for children aged 6 to 8 years. The prevalence of myopia increased 1.4 to 3 times in 2020 compared with the previous 5 years.

**Meaning:**

Home confinement due to coronavirus disease 2019 appeared to be associated with a substantial myopic shift in children; younger (aged 6-8 years) children’s refractive status may be more sensitive to environmental changes than older children, given that they are in an important period for the development of myopia.

## Teaser

This cross-sectional study compares the prevalence of myopia in school-aged children 5 years before the COVID-19 pandemic with the prevalence during home confinement due to the pandemic.

## Introduction

Myopia is a major health issue around the world. The World Health Organization estimates that half of the population of the world may be myopic by 2050.^1,2^ In recent years, insufficient time spent in outdoor activities has been recognized as a major risk factor for myopia development.^3,4^ The duration and intensity of near work activities are also associated with myopia.^5^

In December 2019, a novel coronavirus (severe acute respiratory syndrome coronavirus 2) rapidly spread in China and around the world. In response to the coronavirus disease 2019 (COVID-19) outbreak, the Chinese government started a nationwide school closure as an emergency measure to prevent spreading of the infection at the end of January 2020.^6^ It is estimated that more than 220 million school-aged children and adolescents were confined to their homes; online courses were offered and delivered through the internet. Although these efforts have been shown to control the pandemic in China, concerns have been raised about whether the period of lockdown may have worsened the burden of myopia due to significantly decreased time spent outdoors and increased screen time at home.^7,8,9^ In this study, we aimed to investigate the association of home confinement during the COVID-19 outbreak with myopia development in school-aged children in China.

## Methods

Photoscreenings have been performed annually on children from 10 elementary schools in Shandong, Feicheng, China, since 2015. From 2015 to 2019, students were screened from all grades (grades 1-6, ages 6-13 years) during September, the first month of a new school year. In 2020, schools were closed from January to May due to COVID-19 and reopened in June; the photoscreenings were therefore done during June 2020. The age of children in this study refers to their age on the date of the photoscreening. The study was performed during 6 consecutive years (2015-2020). Data were analyzed in July 2020. This study followed the Strengthening the Reporting of Observational Studies in Epidemiology (STROBE) reporting guideline for cross-sectional studies.

This school-based cross-sectional study was approved by the ethics board of Tianjin Medical University Eye Hospital, Tianjin, China. Written informed consent was obtained before the start of the study from parents of all participants according to the Declaration of Helsinki.^10^ All questions and concerns were addressed before the consent forms were signed. None of the participants were offered compensation or incentives to participate.

### Refraction Screening

Schools were informed of the screening date at least 1 week ahead. Students who routinely wear contact lens were asked to not wear them on the screening day. Students with current corneal refractive therapy (ortho-K) were also asked not to wear the ortho-K lenses the night before the screen date and wear eyeglasses instead on the screen date. The students were asked to remove eyeglasses for the refraction test. No students were receiving low-dose atropine for myopia control in the schools participating in the screening.

Noncycloplegic refractive error was tested using the Spot Vision Screener, version 2.1.4 (Welch Allyn). Testing was conducted by trained staff who obtained results from each child in 3 trials as previously described.^11,12,13^ Briefly, the examiner holds the photoscreener at a 1-m distance from the child. The spherical equivalent refraction (SER) of the child is recorded automatically for both eyes. The measurement range of the screener was limited to ±7.50 diopters (D) in increments of 0.25 D. If the refraction was out of range, ±8.00 D was recorded for further analysis. The software algorithm of the photoscreener would flag a referral for complete eye examination if significant refractive error, anisometropia, or strabismus was detected.^12,13^ Owing to the COVID-19 pandemic, all examiners were trained to perform the 2020 photoscreenings at a safe distance of 1.8 m from the children, with their arms extended to keep the screener at a 1-m distance. All the examiners and children wore masks during the screening. Myopia was defined as an SER of −0.50 D or less. A flowchart for the screening process is shown in eFigure 1 in the Supplement.

Exclusion criteria included wearing contact lenses on the screen day; wearing ortho-K lenses the night before screen day; using eye drops for any kind of ocular diseases, except for simple asthenopia; and having a history of ocular surgery. The examiner questioned the children about the exclusion criteria before testing, and the data were excluded from analysis if any of the situations applied.

### Statistical Analysis

Annual photoscreening data are presented as the mean (SE). The analysis was performed using R, version 4.0.2 software,^14^ and the figures were prepared using RStudio, version 1.3.1056 (RStudio PBC).^15^ Two-proportions z test and 1-way analysis of variance were used when appropriate. Statistical significance was assessed at an unpaired, 2-sided level of *P* < .05.

## Results

A total of 194 904 test results (389 808 eyes) were included in this study, with the children ranging in age from 6 to 13 years. Some children were screened in consecutive years, and thereby their results were included multiple times. The total number of children who were tested was 123 535, with 64 335 boys (52.1%) and 59 200 girls (47.9%). The number of observations for each child ranged from 1 to 6, with a mean (SD) test time of 1.58 (0.87) minutes per child. Sample size, age, and sex distribution are shown in eFigure 2 in the Supplement. There were 62 children with at least 1 eye refraction out of range (<−8.00 D); of these, 21 children had both eye refractions out of range and 41 children had 1 eye refraction out of range. No children had refraction of more than +8.00 D in at least 1 eye.

**Figure 1.**
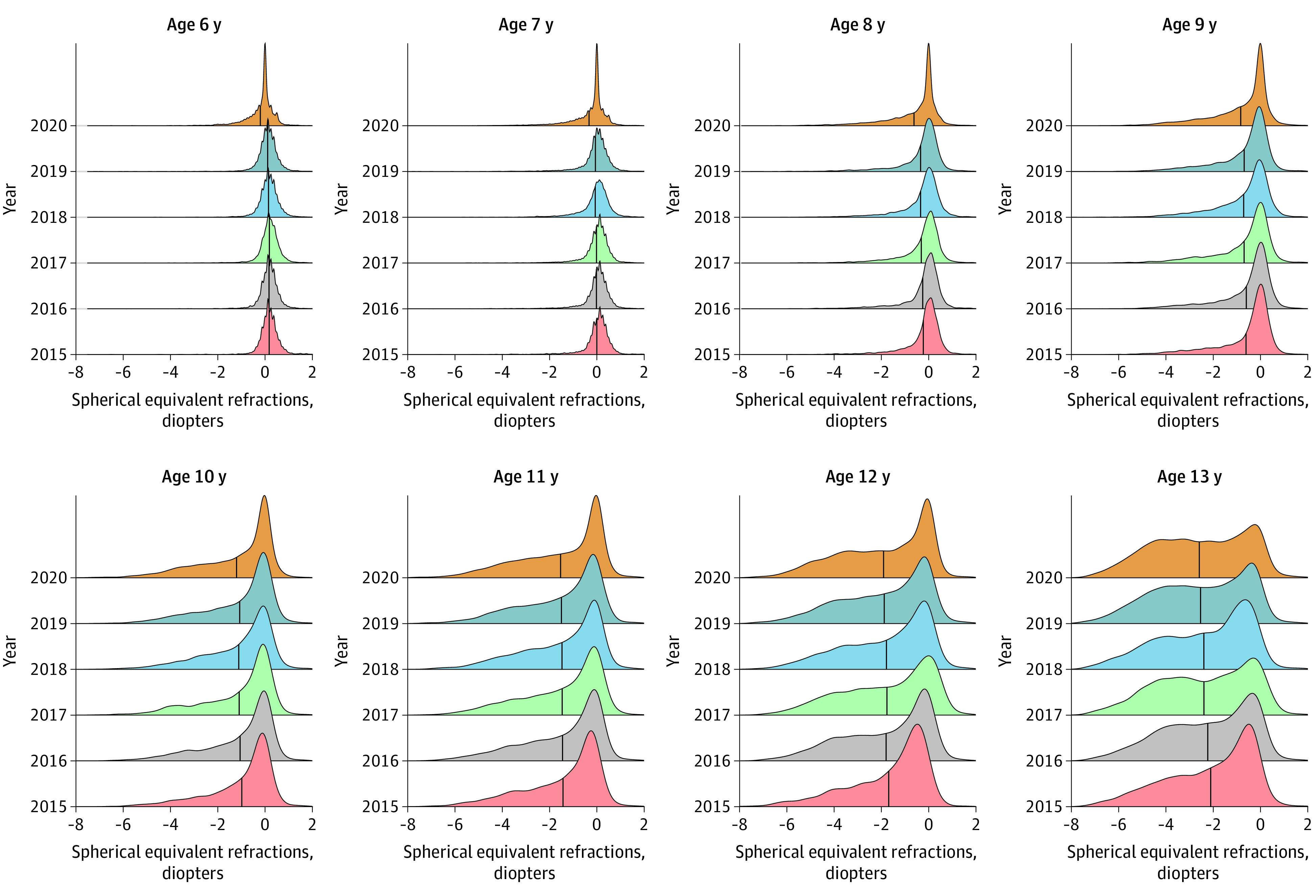
Spherical Equivalent Refraction (SER) Distribution in Primary School Students Aged 6 to 13 Years The number of eyes per year with certain SER is plotted at 6 consecutive years for different age groups (6-13 years). The vertical line in each distribution represents the mean. At age 6 to 8 years, the crest as well as the mean (vertical line) in 2020 were shifted to the left compared with previous years, indicating a myopic shift of SER in 2020. For children aged 9 to 13 years, no clear myopic shift was observed across the 6 years.

The mean SER for each of the 6 screening years is reported in Table 1. We found that, in the annual screenings conducted from 2015 to 2019, the mean SER findings were relatively stable for all age groups. However, the SER was substantially decreased in 2020 compared with previous years (2015-2019), especially for children aged 6 (−0.32 D), 7 (−0.28 D), and 8 (−0.29 D) years (Table 1; Figure 1). The SER differences between 2020 and previous years in children aged 9 to 13 years were −0.12 D for age 9, −0.11 D for age 10, −0.06 D for age 11, −0.05 D for age 12, and −0.05 D for age 13 years. These differences were smaller and not comparable to the larger differences observed in younger children.

**Table 1.** SER Values During Each Year in School-Aged Children

Age, y	No.	SER, mean (SEM)^a^					
		2015	2016	2017	2018	2019	2020
6	22 082	0.21 (0.01)	0.20 (0.01)	0.21 (0.01)	0.18 (0.01)	0.15 (0.01)	−0.17 (0.01)
7	27 979	0.03 (0.01)	0.02 (0.01)	0.02 (0.01)	−0.04 (0.01)	−0.03 (0.01)	−0.31 (0.01)
8	25 877	−0.19 (0.01)	−0.21 (0.01)	−0.28 (0.01)	−0.30 (0.01)	−0.30 (0.01)	−0.59 (0.01)
9	23 591	−0.57 (0.02)	−0.55 (0.01)	−0.65 (0.02)	−0.68 (0.01)	−0.66 (0.01)	−0.80 (0.01)
10	22 910	−0.95 (0.02)	−1.01 (0.02)	−1.04 (0.02)	−1.06 (0.02)	−1.03 (0.02)	−1.17 (0.02)
11	25 373	−1.39 (0.02)	−1.41 (0.02)	−1.43 (0.02)	−1.44 (0.02)	−1.45 (0.02)	−1.51 (0.02)
12	22 742	−1.66 (0.03)	−1.75 (0.02)	−1.71 (0.02)	−1.75 (0.02)	−1.82 (0.02)	−1.87 (0.02)
13	24 350	−2.07 (0.03)	−2.19 (0.02)	−2.35 (0.02)	−2.37 (0.02)	−2.49 (0.02)	−2.54 (0.02)

Abbreviation: SER, spherical equivalent refraction.

^a^ All findings were significant at *P* < .001, with values calculated by 1-way analysis of variance for SERs across 6 years.

**Figure 2.**
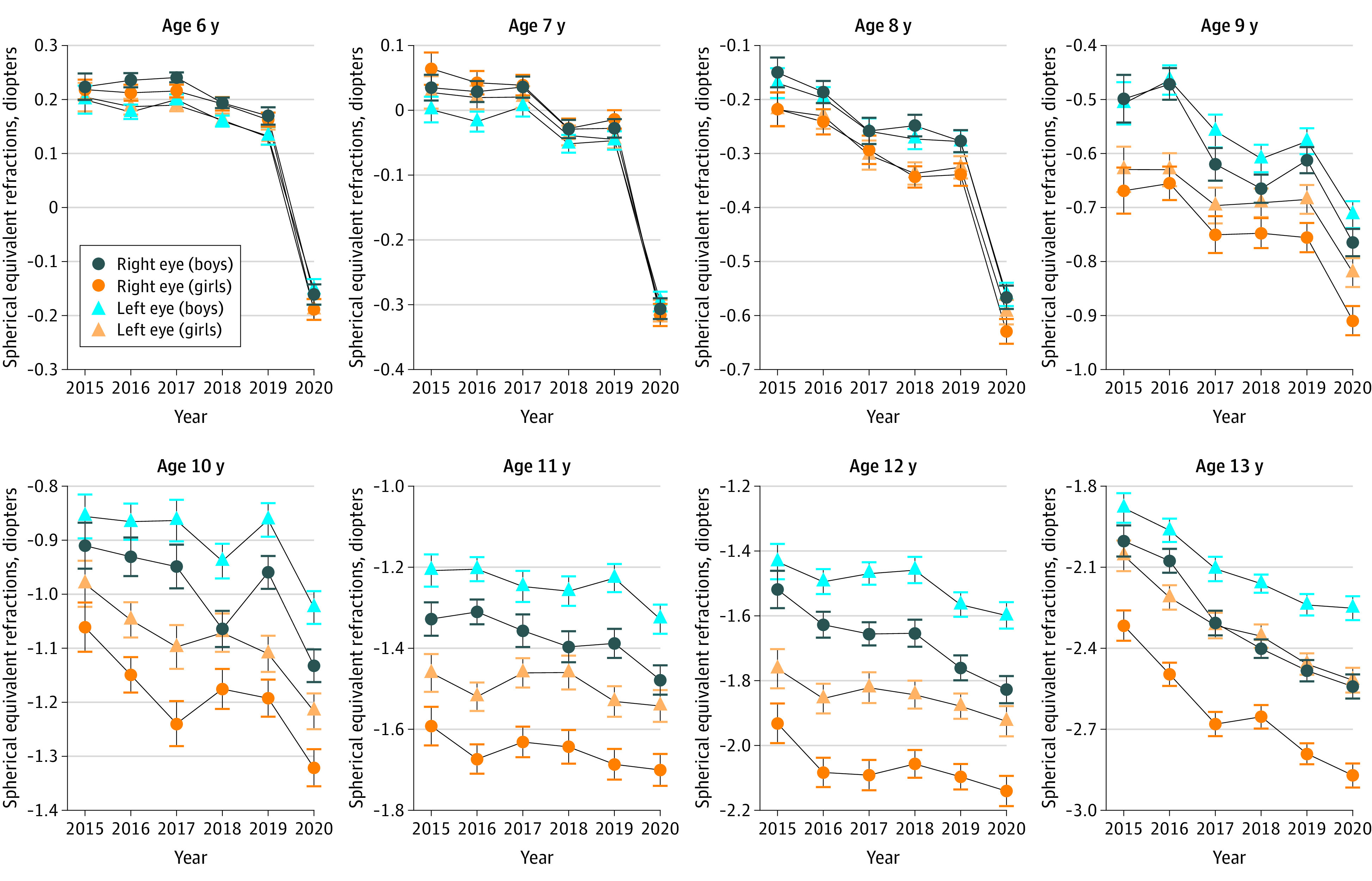
Mean Spherical Equivalent Refraction (SER) for Primary School Students Aged 6 to 13 Years During the 6 Years of Screenings Substantial decreases of SER in 2020 were noted for children aged 6 to 8 years compared with previous years. Overall, girls tended to be more myopic than boys at the same age, while the right eye tended to be more myopic than the left eye. Whiskers indicate SEM.

With further analysis of the data, we found that these changes were observed in both sexes and both eyes (Figure 2). However, the data showed that girls had earlier development of myopia than boys, and the right eye was more myopic than the left eye (Figure 2).

**Figure 3.**
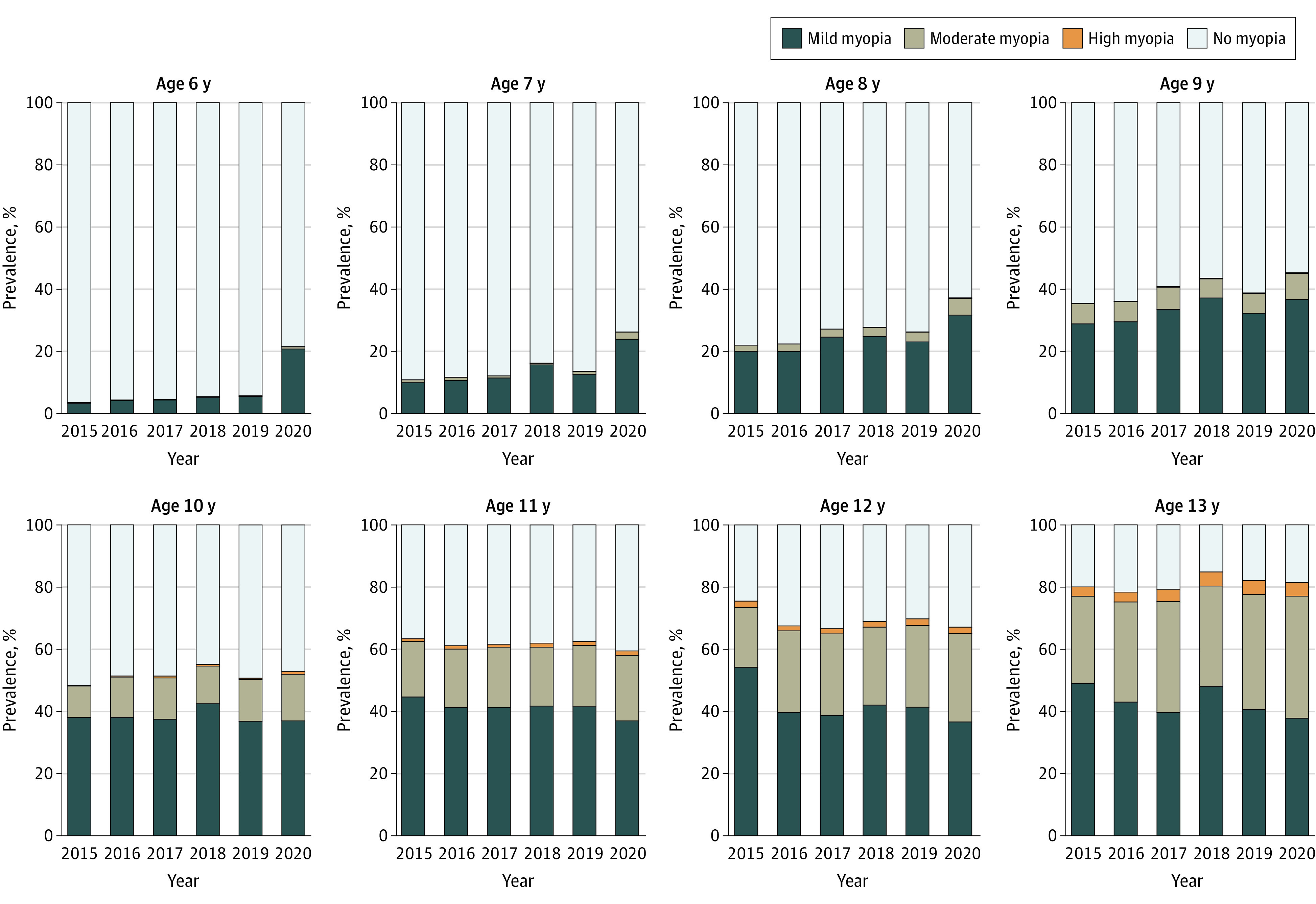
Prevalence of Refractive Error for Primary School Students Aged 6 to 13 Years During the 6 Years of Screenings The prevalence of mild myopia increased in 2020 compared with previous years in children aged 6 to 8 years. Mild myopia: −3 diopters (D) < spherical equivalent refraction (SER) ≤ −0.5 D; moderate myopia: −6 D < SER ≤ −3 D; high myopia: SER ≤ −6 D; and no myopia: SER > −0.5 D.

The myopic shift of SER appeared to be associated with an increase in the prevalence of myopia (SER≤−0.5D) in children aged 6 to 8 years in 2020 compared with previous years. The prevalence of myopia in this age group in 2020 was 21.5% at 6 years, 26.2% at 7 years, and 37.2% at 8 years. These levels were significantly higher than the highest prevalence of myopia in 2015-2019 for these age groups: 5.7% at 6 years in 2019, 16.2% at 7 years in 2018, and 27.7% at 8 years in 2018. (Figure 3; Table 2). Most of the increased cases of myopia were found to be mild (Figure 3).

**Table 2.** Prevalence of Myopia (SER ≤ −0.5D) for Each Year in School-Aged Children

Age, y	No.	Prevalence per year, %						*P* value^a^
		2015	2016	2017	2018	2019	2020	
6	22 082	3.5	4.3	4.5	5.4	5.7^b^	21.5	<.001
7	27 979	10.8	11.7	12.1	16.2^b^	13.6	26.2	<.001
8	25 877	22.0	22.4	27.2	27.7^b^	26.3	37.2	<.001
9	23 591	35.4	36.1	40.8	43.5^b^	38.8	45.3	.09
10	22 910	48.3	51.4	51.3	55.1^b^	50.7	52.8	.03
11	25 373	63.3^b^	61.1	61.6	62.0	62.5	59.5	.004
12	22 742	75.5^b^	67.5	66.6	68.9	69.8	67.1	<.001
13	24 350	80.0	78.4	79.3	84.9^b^	82.1	81.5	<.001

Abbreviation: SER, spherical equivalent refraction.

^a^ *P* value was calculated using 2-proportions *z* test, comparing the prevalence in 2020 with the highest prevalence in the relative age group in 2015-2019.

^b^ Highest prevalence within the age group during the 2015-2019 screening.

Although the prevalence of myopia for children aged 9 years (45.3%) in 2020 was the highest prevalence across the 6 years of measurement, it was not substantially different from the second highest prevalence in 2018 (43.5%). The prevalence of myopia in 2020 ceased to be the highest among the 6 years for children aged 10 to 13 years (Figure 3; Table 2).

## Discussion

This school-based photoscreening project was initiated in 2015 to monitor the prevalence of myopia among children in Feicheng, China. The Spot Vision Screener used in this study has been shown to have good consistency with the cycloplegic refraction test, which makes it a reliable screening tool for myopia.^11,12,16,17,18,19^ Compared with cycloplegic refraction, the results from the Spot Vision Screener showed an overall overestimation of myopia, with a range of −0.17 to −0.49 D in children aged 3 to 10 years.^12,19,20,21^ The features of noncontact and 1-m distance of the screener allowed us to safely continue this project during this COVID-19 pandemic. Although the photoscreenings usually take place in September, when the new school year begins, the COVID-19 pandemic led to a nationwide school closure in China from January to May 2020. Students were confined to their homes until June when the schools reopened. Therefore, the photoscreenings in 2020 took place during June.

In this study, the SER distribution in this population appeared to be stable from 2015 to 2019, with a slight overall myopic shift (Figure 1 and Figure 2). However, in the 2020 screenings, there was a substantial myopic shift (approximately −0.3 D) for children aged 6 to 8 years. Given the large sample sizes, although statistical significance was also shown for myopic shifts in children aged 9 to 13 years, we do not consider those shifts to be of clinical significance. The SER difference between 2020 and the earlier years in children aged 9 to 13 years was approximately −0.1 D, which is smaller than the difference observed in children aged 6 to 8 years. Very large sample sizes tend to decrease *P* values toward 0. Solely relying on *P* values can lead the researcher to claim support for results of limited to no clinical significance.^22^

Due to COVID-19 in 2020, the school-aged children were confined to their homes from January to May, and online courses were offered. For the screened population, their daily online course hours for grades 1 and 2 is 1 hour and the time for grades 3 to 6 is 2.5 hours. Children’s indoor activities and screen time therefore increased and their outdoor activities were decreased, often to none.^6^ Lessened outdoor activity is known to be significantly associated with a higher incidence of myopia in school-aged children.^1,3,5^ Concerns have been raised about whether home confinement may worsen the burden of myopia.^7,8,9^ To our knowledge, we provide the first evidence that the concern may be justified, especially for younger children aged 6 to 8 years. If home confinement is necessary, parents should control the children’s screen time as much as possible and increase the allowable outdoor activity while maintaining safe social distancing.

This substantial myopic shift was not seen in any other year-to-year comparison, making the cause possibly due to the unusual occurrence of home confinement in 2020. However, when comparing the SER and myopia prevalence in 2020 with previous years, other factors were also noted. First, as mentioned above, the 2020 screening took place in June rather than the usual screening time of September. We believe that the SER obtained right after home confinement ended in June reflects the outcome of home confinement. Second, the screening process differed in 2020 from that in previous years, because all children wore masks and the examiners held the photoscreener with their arms extended to keep an adequate social distance. However, we believe these 2 factors could not explain the findings in this study, given that these factors applied equally to all ages. Assuming they are influential, myopic shifts would be shown in children of all ages examined, but not solely in those aged 6 to 8 years. Moreover, to our knowledge, there has been no report showing that mask wearing and/or the season in which screening takes place could be associated with the results of a refraction test.

In this study, the prevalence of myopia appeared to be approximately 3 times higher in 2020 than in other years for children aged 6 years, 2 times higher for children aged 7 years, and 1.4 times higher for those aged 8 years (Table 2). Such a substantial increase in the prevalence of myopia was not seen in the older age groups (9-13 years), despite the fact that the older children (grades 3-6) were offered more intense daily online learning courses (2.5 hours) compared with the younger students (grades 1-2, 1 hour daily). These findings led us to a hypothesis that younger children are more sensitive to the environmental change than older children. In the setting of this specific study, the period of environmental change (home confinement) was 4 months and the children who appeared to be affected were aged 6 to 8 years.

There were factors that we were unable to evaluate in this study, including the adherence to school offerings, the exact amount of near work or screen time, and the exact number of daily outdoor activity hours for each child. The lack of such information could limit the interpretation of the results of this study. However, given the fact that the younger children were assigned fewer online learning tasks than the older ones, it is unlikely that rapidly progressing myopia in younger children was caused by more intense screen time or near work. Moreover, all children were restricted at home and there is no reason to believe that outdoor activity time was the cause of such differences.

It is unknown whether older children (aged 9-13 years) would be more myopic if they undergo a longer period of home confinement. If that is the case, the period of environmental change may be the main risk factor for myopia development, with the younger children more sensitive to the environmental change than the older children. Children aged 6 to 8 years may be experiencing an important period for myopia development. Within this age window, the plasticity of myopia is high and myopia control may be easier. Beyond this age window, the plasticity of myopia is low and myopia is harder to control during environmental changes. This idea is supported by the study of VanderVeen et al,^23^ which reported that orthokeratology may be effective in slowing myopic progression in children and adolescents, with a potentially greater effect when initiated at an early age (6-8 years).

Among the children in the present study, it seems logical that the more-affected younger children were experiencing an important period of myopia development—a time of high plasticity. This potential requires further evaluation within other populations. If there is such an important period for myopia development, strategies for myopia control and intervention could be implemented with a possibly global influence.

In this study, girls were found to have earlier myopia development than boys (Figure 2). Similar sex differences in myopia development have also been seen in other studies.^11,24^ In a 13-year series of population-based prevalence surveys, female sex was found to be a statistically significant risk factor for myopia (odds ratio, 1.24; 95% CI, 1.21-1.27).^25^ Other multicenter or comparative studies have shown that girls had steeper corneas, shallower anterior chambers, steeper lens powers, and shorter axial length than boys.^26,27^ These differences between sexes appeared around the age of 8 years in the present study (Figure 2). The reason is not fully understood. Some believe that the sex differences in the early adolescent years may be associated with different ages of onset of puberty and estrogen level changes.^28,29^ Another finding from this study is that the right eye was often more myopic than the left eye, starting at approximately age 9 years (Figure 2). This disparity of SER between eyes was also seen in another population-based photoscreening study^11^ and is believed to be associated with the development of anisometropia in children.^11,30,31^ However, much remains to be known regarding this intereye difference. Studies have reported that the dominant eye may have a greater degree of myopia than the nondominant eye in individuals with anisometric myopia.^32,33^ To our knowledge, no significant association has been found between hand dominance and ocular dominance.^32,34^

Is this myopic shift temporary or permanent? Is it reversible? Our group intends to follow-up on the same population and continue the school-based annual photoscreening. With the results of the 2021 school year photoscreening, we shall have more information to address these questions.

### Limitations

This study has limitations. First, preschool-aged children were not included; it would be useful to know how the younger children responded to the environmental change. Second, the photoscreener provides only noncycloplegic refraction results. It is useful in population-based screening and myopia monitoring with high test accuracy but currently is not considered a substitute for cycloplegic refraction. Third, previous use of orthokeratology may cause corneal change in the curvature as well as the epithelium,^23,35^ which may affect the refraction test results and introduce bias to this study. However, given the fact that the area we screened is relatively underdeveloped and orthokeratology is not popular, the potential affect on this large sample size study is low. Fourth, information on the ocular biometry, such as axial length and cornea curvature, was not available for this study. In school-aged children, the development of myopia is mostly due to excessive ocular axial elongation, with relatively stable corneal changes.^26,36,37^ Throughout childhood, the association between the amount of myopia and the axial length is nonlinear and nonconstant.^26^ It was recently reported that refractive error variance in school-aged children was best explained by variation in the axial length/corneal radius ratio, with higher values associated with more myopic refraction.^38^ Lacking such data has limited the power of this study in analyzing the underlying biological processes for the rapid progression of myopia in younger children compared with older children. Fifth, with graduation and new enrollment every year, the annually tested populations differ from year to year, and so the differences reflect population means.

## Conclusions

The findings of this study suggest that home confinement during the COVID-19 pandemic was associated with a substantial myopic shift for younger school-aged children (6-8 years) according to the 2020 school-based photoscreenings. Younger children’s refractive status may be more sensitive to environmental changes than older children, given the younger individuals are in an important period for the development of myopia. Further studies are needed to assess the generalizability of these findings and the long-term follow-up of these children.

## Notes

Supplement. **eFigure 1.** Flow Chart for Screening Process **eFigure 2.** Histogram of Sample Size, Age, and Gender Distribution by Year
