# Supplementary material for: Progression of Myopia in School-Aged Children After COVID-19 Home Confinement
Source: JAMA Ophthalmol. 2021 Jan 14;139(3):293–300. doi: 10.1001/jamaophthalmol.2020.6239 (PMC7809617; doi:10.1001/jamaophthalmol.2020.6239)
Supplement: Supplement. — eFigure 1. Flow Chart for Screening Process eFigure 2. Histogram of Sample Size, Age, and Gender Distribution by Year [file jamaophthalmol-e206239-s001.pdf]

Supplementary Online Content

Wang J, Li Y, Musch DC, et al. Progression of myopia in school-aged children after COVID-19 home confinement. *JAMA Ophthalmol*. Published online January 14, 2021. doi:10.1001/jamaophthalmol.2020.6239

eFigure 1. Flow Chart for Screening Process

eFigure 2. Histogram of Sample Size, Age, and Gender Distribution by Year

This supplementary material has been provided by the authors to give readers additional information about their work.

© 2021 Wang J et al. *JAMA Ophthalmology*.

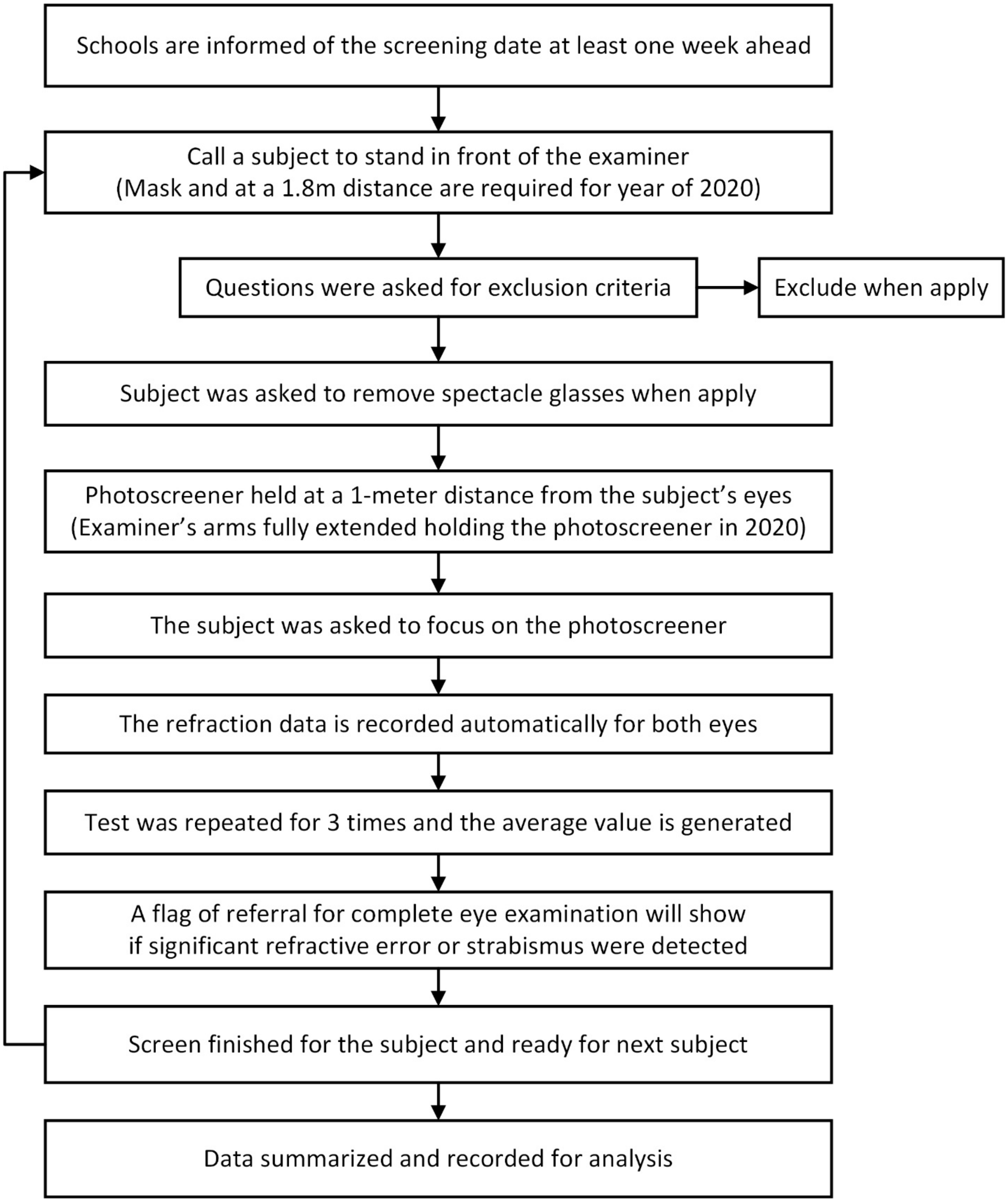

Figure 1. Flow Chart for Screening Process

© 2021 Wang J et al. *JAMA Ophthalmology*.

eFigure 2. Histogram of Sample Size, Age, and Gender Distribution by Year

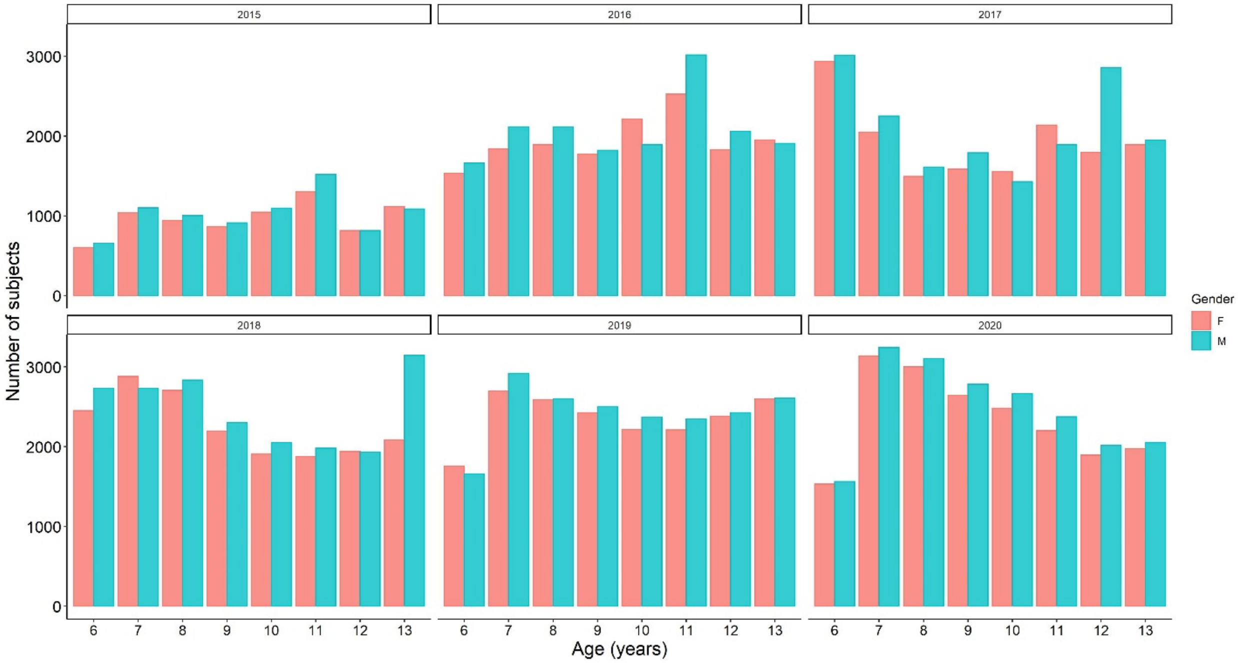

© 2021 Wang J et al. *JAMA Ophthalmology*.
